# Clomiphene and Its Isomers Block Ebola Virus Particle Entry and Infection with Similar Potency: Potential Therapeutic Implications

**DOI:** 10.3390/v8080206

**Published:** 2016-08-02

**Authors:** Elizabeth A. Nelson, Alyson B. Barnes, Ronald D. Wiehle, Gregory K. Fontenot, Thomas Hoenen, Judith M. White

**Affiliations:** 1 Department of Cell Biology, University of Virginia, Charlottesville, VA 22908, USA; en2b@virginia.edu (E.A.N.); abb9uu@virginia.edu (A.B.B.); 2 Repros Therapeutics, Inc., The Woodlands, TX 77380, USA; rwiehle@reprosrx.com (R.D.W.); GFontenot@reprosrx.com (G.K.F.); 3 Laboratory of Virology, Division of Intramural Research, National Institutes of Health, Hamilton, MT 59840, USA; thomas.hoenen@fli.bund.de; 4 Friedrich-Loeffler-Institut, D-17493, Greifswald-Insel Riems, Germany

**Keywords:** Ebola, filovirus, enclomiphene, zuclomiphene, anti-viral, Ebola Virus disease, Ebola virus survivors

## Abstract

The 2014 outbreak of Ebola virus (EBOV) in Western Africa highlighted the need for anti-EBOV therapeutics. Clomiphene is a U.S. Food and Drug Administration (FDA)-approved drug that blocks EBOV entry and infection in cells and significantly protects EBOV-challenged mice. As provided, clomiphene is, approximately, a 60:40 mixture of two stereoisomers, enclomiphene and zuclomiphene. The pharmacokinetic properties of the two isomers vary, but both accumulate in the eye and male reproductive tract, tissues in which EBOV can persist. Here we compared the ability of clomiphene and its isomers to inhibit EBOV using viral-like particle (VLP) entry and transcription/replication-competent VLP (trVLP) assays. Clomiphene and its isomers inhibited the entry and infection of VLPs and trVLPs with similar potencies. This was demonstrated with VLPs bearing the glycoproteins from three filoviruses (EBOV Mayinga, EBOV Makona, and Marburg virus) and in two cell lines (293T/17 and Vero E6). Visual problems have been noted in EBOV survivors, and viral RNA has been isolated from semen up to nine months post-infection. Since the clomiphene isomers accumulate in these affected tissues, clomiphene or one of its isomers warrants consideration as an anti-EBOV agent, for example, to potentially help ameliorate symptoms in EBOV survivors.

## 1. Introduction

The epidemic of Ebola virus (EBOV) that swept through Western Africa beginning in December 2013, was the worst outbreak of this deadly hemorrhagic fever virus in recorded history. Over 28,000 individuals were infected, over 11,000 died, and there are currently an estimated 16,000 survivors [1,2]. In addition to the tragic loss of life, many survivors are now suffering from sequelae that arose after they recovered from their EBOV infections; these sequelae include visual problems, hearing loss, body aches, severe fatigue, and memory loss [3,4]. Moreover, infectious EBOV has been shown to persist in semen for six months, EBOV RNA has been found in semen as late as nine months post-infection, and at least one case of sexual transmission has been reported [5,6].

Currently there are no approved drugs with which to treat EBOV patients. Consequently, during the recent outbreak several agents were administered to patients on a compassionate care basis; these included the ZMapp cocktail of monoclonal antibodies [7,8], hyperimmune serum [9,10], and several low molecular weight anti-viral drugs [11,12,13]. Additional therapeutic antibodies [14,15,16,17] have recently been described, and two novel low molecular weight viral RNA polymerase inhibitors are in development [18,19]. A different approach has been to screen U.S. Food and Drug Administration (FDA)-approved drugs for anti-EBOV activity, with the goal of repurposing an approved drug, or combination of approved drugs, for use as a prophylactic and/or therapeutic in the advent of a future outbreak [20,21,22,23,24]. Clomiphene surfaced in two independent screens of FDA-approved drugs for anti-EBOV activity [20,21,22]; it blocks EBOV entry and infection in tissue culture cells and provides up to 90% protection in the mouse model of EBOV disease [20,21]. Clomiphene is a selective estrogen receptor modulator that is used to treat female infertility due to anovulation [25,26,27]. It was also proposed in 1991 as a drug to reverse impotence in men due to low testosterone levels [28], and is currently used off-label for men with hypogonadism. We found, however, that clomiphene exerts its anti-EBOV activity in cell cultures irrespective of expression of the estrogen receptor alpha and/or beta subunits [20]. Instead, clomiphene likely exerts its anti-EBOV activity because it is a cationic amphiphilic drug [29] that accumulates in endolysosomes, the site of EBOV fusion and cytoplasmic entry [30,31,32]. Similar to other cationic amphiphilic drugs [29,33], clomiphene blocks a late step in the complex process by which the EBOV genome gains access to the host cell cytoplasm to initiate replication, most likely by blocking fusion between the EBOV and endolysosomal membranes [29].

Clomiphene citrate, the clinically-administered drug and the formulation tested to date for anti-EBOV activity, is a mixture of two diastereoisomers: ~60% *trans*-clomiphene (enclomiphene) and ~40% *cis*-clomiphene (zuclomiphene). Whereas enclomiphene exhibits high anti-estrogenic properties, zuclomiphene does not [34,35,36]. The two isomers also differ in other pharmacokinetic properties. For examples, zuclomiphene has a longer half-life in serum [37,38] and persists in most tissues for longer periods of time than enclomiphene [39]. It could, therefore, be proposed that zuclomiphene is the major contributor to adverse side effects seen in individuals given clomiphene citrate and, conversely, that enclomiphene would impart fewer side effects [39,40]. Due to these features, as well as its ability to raise testosterone levels, enclomiphene is currently in clinical development as a treatment for secondary hypogonadism (low testosterone levels) [41].

Because of the differing pharmacological features of enclomiphene and zuclomiphene and the ongoing clinical development of enclomiphene, we compared the effects of clomiphene and its isomers on EBOV entry and replication. We found that clomiphene, enclomiphene, zuclomiphene, and two of their primary metabolites (4-hydroxy-enclomiphene and 4-hydroxy-zuclomiphene) block EBOV entry and replication to similar levels. Compared to clomiphene, enclomiphene, and zuclomiphene displayed similar dose profiles for blocking EBOV entry and replication, as tested in two cell types and with the glycoproteins from two isolates of EBOV as well as from a Marburg virus.

## 2. Materials and Methods

### 2.1. Compounds

Clomiphene, enclomiphene, zuclomiphene, 4-hydroxy-enclomiphene, and 4-hydroxy-zuclomiphene were supplied by Repros Therapeutics Inc. (The Woodlands, TX, USA). Compound stocks (5 mM) were prepared in dimethyl sulfoxide (DMSO) and stored at room temperature until use.

### 2.2. Cells

HEK 293T/17 cells were obtained from the University of Virginia Tissue Culture Facility (ATCC CRL-11268; Charlottesville, VA, USA) and Vero E6 cells (ATCC CRL-1586) were maintained in growth medium (high glucose Dulbecco's Modified Eagle Medium (DMEM)) supplemented with 1% l-glutamine, 1% sodium pyruvate, and 1% antibiotic/antimycotic, all from Gibco Life Technologies (University of Virginia Tissue Culture Facility, Charlottesville, VA, USA). HEK 293T/17 and Vero E6 cells contained either 10% supplemented calf serum (SCS; Hyclone, ThermoFisher Scientific, Waltham, MA, USA) or 10% fetal bovine serum (FBS; Seradigm, University of Virginia Tissue Culture Facility, Charlottesville, VA, USA), respectively.

### 2.3. Preparation of EBOV trVLPs and Entry Reporter VLPs

Transcription/replication-competent viral-like particles (trVLPs) were prepared (under biosafety level 2 (BSL2) conditions) as described in [42,43]. Briefly, to prepare a p0 stock, 50% confluent HEK 293T/17 cells, seeded 24 h prior in six well plates, were transfected with pCAGGS-NP, pCAGGS-VP35, pCAGGS-VP30, pCAGGS-L, a tetracistronic minigenome plasmid, and pCAGGS-T7 polymerase using TransIT-LT1 (Mirus, Madison, WI, USA). The minigenome plasmid encodes *Renilla* luciferase, as well as the matrix proteins VP40 and VP24 and the GP envelope protein from EBOV. 24 h post transfection, the medium in each well was replaced with 4 mL fresh growth medium containing 5% FBS. 72 h after transfection, the medium (containing trVLPs harboring the *Renilla* luciferase-containing mini-genome) was harvested, pooled, and cleared of cellular debris by centrifugation for 5 min at 800× *g*. p1 and p2 stocks were prepared by serial passaging of p0 stocks on HEK 293T/17 target cells freshly transfected with pCAGGS-NP, pCAGGS-VP35, pCAGGS-VP30, pCAGGS-L, and pCAGGS-Tim1. trVLPs (p0, p1, and p2) were stored on ice and used within two weeks.

Entry reporter viral-like particles (VLPs) bearing GPs from Mayinga and Makona EBOV, and from Marburg virus (MARV; Angola isolate, gift of Dr. Christopher Broder (Uniformed Services University of the Health Sciences, Bethesda, MD, USA) were prepared essentially as described in previous work [20,21,29,30]. In brief, 80% confluent HEK 293T/17 cells were transfected with cDNAs encoding EBOV or MARV GP, VP40, mCherry-VP40, and β-lactamase-VP40 (βlam-VP40) (although not utilized in this study, mCherry-VP40 in the VLPs can be used to monitor VLP binding to and trafficking within cells as described in [29,30]). The cell medium was collected 24 and 48 h post-transfection and was cleared of cellular debris. VLPs in the cleared medium were pelleted through a 20% sucrose cushion by centrifugation, resuspended in HM buffer (20 mM HEPES, 20 mM MES, 130 mM NaCl, pH 7.4), and repelleted. The final VLP pellet was resuspended (1:100 starting volume of medium) in 10% sucrose-HM. The total protein concentration of the VLPs was determined by bicinchoninic acid (BCA) assay. All entry-reporter VLP preparations were assessed by western blot analyses (for the presence of the indicated glycoprotein as well as EBOV VP40) and titered on Vero E6 cells to confirm entry competency. Entry-reporter VLPs were frozen in single use aliquots at −80 °C and were used within four months.

### 2.4. trVLP Infection and Parallel Cell Viability Assays

Infection of HEK 293T/17 cells by trVLPs was assayed as described [42,43] with minor modifications. In brief, to prepare target cells, HEK 293T/17 cells were seeded in parallel opaque white 96-well plates (BD Falcon, ThermoFisher Scientific, Waltham, MA, USA) for trVLP and cell viability assays and in clear-bottom 96-well plates to assess cell density and transfection efficacy. At 18–24 h post seeding, when the cells were approximately 50% confluent, the cells were transfected with (per well) 6.94 ng pCAGGS-NP, 6.94 ng pCAGGS-VP35, 4.16 ng pCAGGS-VP30, 55.55 ng pCAGGS-L, and 13.88 ng pCAGGS-Tim1 using 0.3 μL TransIT-LT1. These plasmids are necessary for the target cells to support entry and replication of incoming trVLPs. To assess transfection efficacy and to provide a negative control, wells on each plate were transfected as above, but with a green fluorescent protein (GFP) expression plasmid in place of pCAGGS-L. 18–24 h post transfection, the medium was removed and these target HEK 293T/17 cells were pre-treated with the indicated concentration(s) of the indicated drug(s) (DMSO for mock) diluted in Opti-MEM1 (OMEM, Gibco Life Technologies via University of Virginia Tissue Culture Facility, Charlottesville, VA, USA) for 1 h at 37 °C in a 5% CO_2_ incubator.

To assess trVLP infection, the pretreatment solution was removed and replaced with 25 μL or 50 μL trVLPs diluted to 200 μL in growth medium containing 10% SCS and the indicated concentration(s) of the indicated drug(s) (DMSO for mock). The cells were then incubated for 48 h at 37 °C in a 5% CO_2_ incubator, after which the medium was replaced with 40 μL of fresh growth medium containing 10% SCS. 40 μL of RenillaGlo substrate (Promega, Madison, WI, USA) was then added to each well and the plate was immediately analyzed on a GloMax plate reader.

To assess cell viability, the pretreatment solution was removed and replaced with 200 μL fresh growth medium containing 10% SCS and the indicated concentration(s) of the indicated drug(s) (DMSO for mock). The cells were then incubated for 48 h at 37 °C in a 5% CO_2_ incubator, after which the medium was replaced with 40 μL of fresh growth medium containing 10% SCS. 40 μL of CellTiter-Glo 2.0 (Promega) was then added to each well and the plate placed on a Jitterbug orbital shaker (Boekel Scientific, ThermoFisher Scientific, Waltham, MA, USA) set at 575 rpm for 2 min at room temperature (RT). The plate was then incubated at RT for 10 min, after which the luminescent signal was detected using a Synergy HT (BioTek, Winooski, VT, USA) plate reader.

### 2.5. VLP Entry and Parallel Cell Viability Assays

VLP entry assays were performed as described [20,29,30] with minor modifications. In brief, 25–50,000 Vero E6 or 293T/17 cells were seeded per well of a clear 96-well plate. At 18–24 h post seeding, when the cells were ~80%–90% confluent, the cells were treated with the indicated drug(s) at the indicated concentration(s) (DMSO for mock) diluted in OMEM for 1 h at 37 °C in a 5% CO_2_ incubator. VLPs diluted in OMEM in the presence of the indicated drug(s) at the indicated concentration(s) (DMSO for mock) were bound to the cells by spinfection (250× *g*) for 1 h at 4 °C. The cells were then incubated for 3 h in a 37 °C, 5% CO_2_ incubator. The βlam substrate CCF2-AM (Life Technologies, ThermoFisher Scientific, Waltham, MA, USA) was then loaded into the cells as previously described, but with 20 mM instead of 5 mM Probenecid (MP Biomedicals, ThermoFisher Scientific, Waltham, MA, USA) in the loading buffer. The cells were incubated overnight at RT and then fixed and analyzed by flow cytometry as described [20,29,30].

To measure cell viability, 25–50,000 Vero E6 cells, seeded and grown as above but in 96-well opaque white plates were treated (as above) for VLP entry, but VLPs were not added and CCF2-AM was not loaded. After the overnight incubation at RT (see above), the medium was removed from the cells and replaced with 50 μL of fresh medium per well. 50 microliters (per well) of CellTiter‑Glo 2.0 was then added. The plate was placed on a Jitterbug orbital shaker (575 rpm) for 2 min at RT. The plate was then incubated at RT for 10 min, after which the luminescent signal was detected using a BioTek Synergy HT plate reader.

### 2.6. Analysis of Eight-Point Dose Response Data

For both trVLP infection and VLP entry, raw data were normalized to mock infection/entry signals, and percent inhibition of infection/entry was determined. GraphPad Prism 7.0 was used to perform non-linear regression analyses (log (agonist) vs. response; variable slope) of the percent inhibition values. Inhibitory concentration, 50% (IC_50_) and 90% (IC_90_) values were interpolated based on the regression curve, and EC_50_ values were automatically generated. The GraphPad program “ECanything” [44] was used to determine EC_90_ values, which are based on the EC_50_ and hill slope values from the regression analysis.

## 3. Results

### 3.1. The Two Clomiphene Isomers and Their Primary Metabolites Block EBOV Entry and Replication

**Figure 1:**
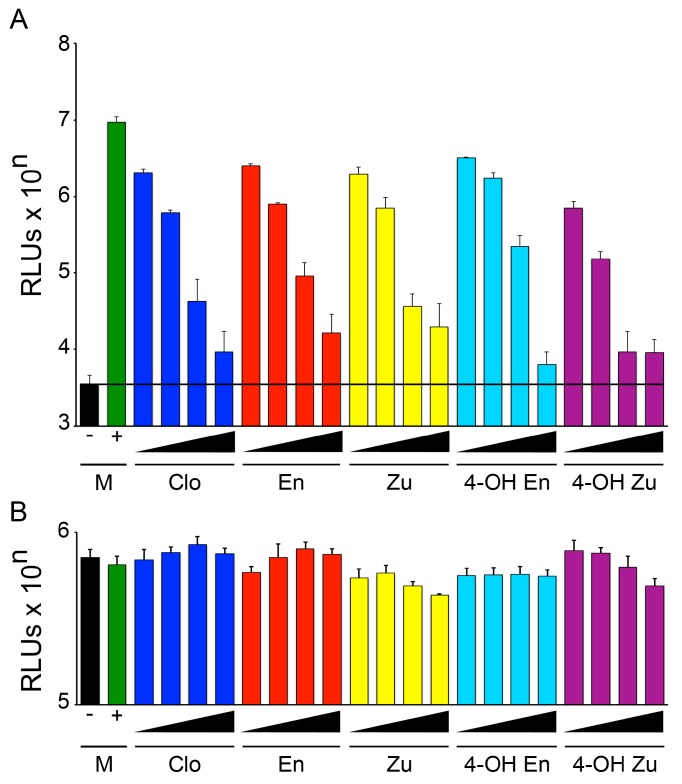
Clomiphene as well as its isomers and metabolites inhibit Ebola virus (EBOV) transcription/replication-competent viral like particle (trVLP) infection with no detectable effect on cell viability. (**A**) HEK293T/17 (target) cells were transfected with plasmids encoding the EBOV polymerase (EBOV L (+)), or green fluorescent protein (GFP) (−), as well as EBOV nucleoprotein (NP), EBOV VP30, EBOV VP35, and T-cell immunoglobulin and mucin domain 1 (Tim1). After 18-24 h, the cells were treated with dimethyl sulfoxide (DMSO) (Mock, M) or with increasing concentrations (1, 2.5, 5, or 10 μM) of clomiphene (Clo), enclomiphene (En), zuclomiphene (Zu), 4-hydroxy-enclomiphene (4-OH En) or 4-hydroxy-zuclomiphene (4-OH Zu) for 1 h at 37 °C. The cells were then infected in the presence of the indicated drug with 25 μL trVLPs and assayed 72 h later for *Renilla* luciferase activity. Data are averages of triplicate samples (cells only background subtracted). The black bar and line indicate background signal (-, cells transfected with GFP in place of EBOV L); and (**B**) parallel HEK293T/17 cells were treated as in (**A**) but mock infected with 25 μL medium instead of trVLPs. After 72 h at 37 °C, cell viability was assayed using CellTiter Glo 2.0. Data are averages of triplicate samples. Error bars represent standard deviation (SD) relative to Mock (DMSO, +EBOV L) treated samples. trVLP infection was significantly inhibited by all concentrations of all drugs tested (*p* ≤ 0.01 for 1 μM En, 1 μM Zu, 1 μM 4-OH En; *p* ≤ 0.001 for all others). No significant inhibition of cell viability was observed. Similar results were seen in an additional experiment.

Clomiphene is a ~60:40 mixture of two stereoisomers: enclomiphene and zuclomiphene. Their primary metabolites are 4-hydroxy-enclomiphene and, to a lesser extent, 4-hydroxy-zuclomiphene. We first compared the ability of clomiphene, enclomiphene, zuclomiphene, 4-hydroxy-enclomiphene, and 4-hydroxy-zuclomiphene to block EBOV using the trVLP life cycle modeling system described by Watt and Hoenen [42,43]. As seen in Figure 1A, both isomers of clomiphene (enclomiphene and zuclomiphene) as well as the 4-hydroxy metabolites of each (4-hydroxy-enclomiphene, and 4-hydroxy-zuclomiphene) displayed dose-dependent inhibition of trVLP infection similar to that seen with the parent mixture (clomiphene) when cells were pretreated for 1 h with increasing concentrations of compound. At a dose of 5 μM, all five forms of clomiphene showed approximately equal potency, reducing the trVLP (*Renilla* luciferase) signal by approximately 1000-fold. Parallel sets of cells showed no evidence of cytotoxicity by any form of clomiphene (Figure 1B).

**Figure 2:**
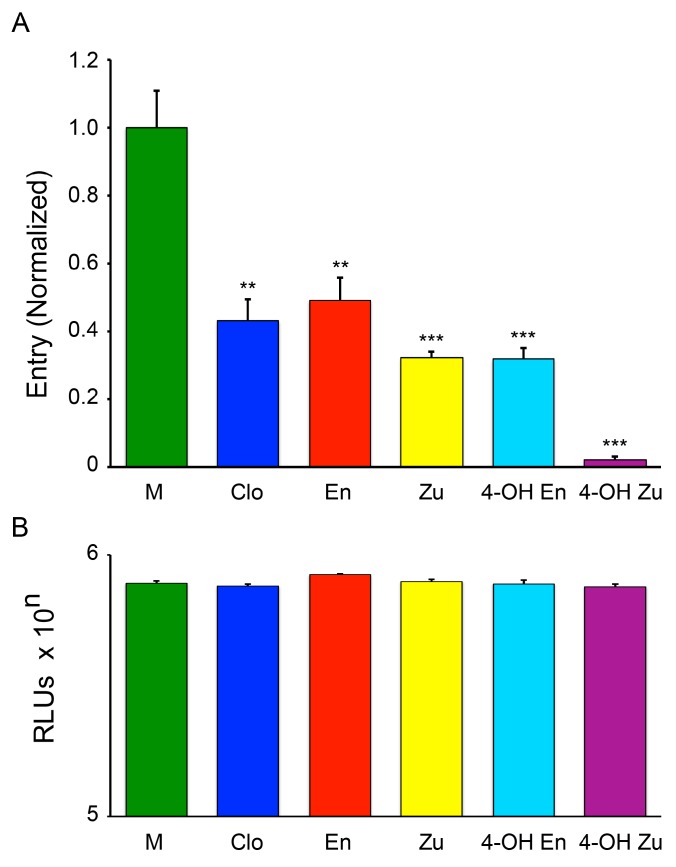
Clomiphene, its isomers, and metabolites inhibit EBOV VLP entry with no effect on cell viability. (**A**) Vero E6 cells were treated with 5 μM clomiphene (Clo), enclomiphene (En), zuclomiphene (Zu), 4-hydroxy-enclomiphene (4-OH En), 4-hydroxy-zuclomiphene (4-OH Zu), or DMSO (mock, M) for 1 h at 37 °C. VLPs bearing EBOV GP (Mayinga strain) were then bound in the presence of the indicated drug (5 μM) or DMSO (mock). After further incubation at 37 °C (3 h), VLP entry was assayed as described in section 2.5. Data are averages of triplicate samples normalized to the average % entry for mock treated controls (20.7%). Error bars represent SD. Similar results were observed in two additional experiments (**B**) Parallel Vero E6 cells were treated with 5 μM of the indicated drug or DMSO (mock) as in (**A**). After 3 h at 37 °C, cell viability was determined as described in the Methods. Data are averages of triplicate samples ± SD. Asterisks indicate significance relative to mock (DMSO) treated samples (** *p* ≤ 0.01, *** *p* ≤ 0.001).

Our previous findings indicated that clomiphene blocks EBOV infection by blocking entry of viral particles into the cell cytoplasm [20,29]; this effect appears to be at the level of fusion between the membrane of the viral particle and the limiting membrane of an Niemann-Pick disease, type C1 positive (NPC1^+^) endolysosome, the site of EBOV fusion [30,31,32]. We, therefore, compared the effects of clomiphene, enclomiphene, zuclomiphene, 4-hydroxy-enclomiphene, and 4-hydroxy-zuclomiphene on EBOV entry using entry reporter VLPs, as described previously [20,29,30]. At a concentration of 5 μM, clomiphene, both of its isomers (enclomiphene and zuclomiphene), as well as the 4-hydroxy metabolite of each isomer blocked VLP entry (Figure 2A), with no evidence of cytotoxicity (Figure 2B). In this system, 4-hydroxy-zuclomiphene appeared more potent than the other forms of clomiphene (Figure 2A), but this is likely an assay-dependent result, as 4-hydroxy-zuclomiphene was not more potent in the trVLP assay (Figure 1A).

### 3.2. Clomiphene, Enclomiphene, and Zuclomiphene Inhibit EBOV trVLP Infection and VLP Entry with Equal Potency

As elaborated in sections 1 and 4, enclomiphene and zuclomiphene exhibit different pharmacological properties. Notably, zuclomiphene has a longer half-life [37,45], and evidence suggests that the zuclomiphene component is responsible for more of the adverse side effects seen in mice treated with clomiphene [40]. We, therefore, compared clomiphene, enclomiphene, and zuclomiphene in more detail for their effects on trVLP infection and VLP entry.

**Figure 3:**
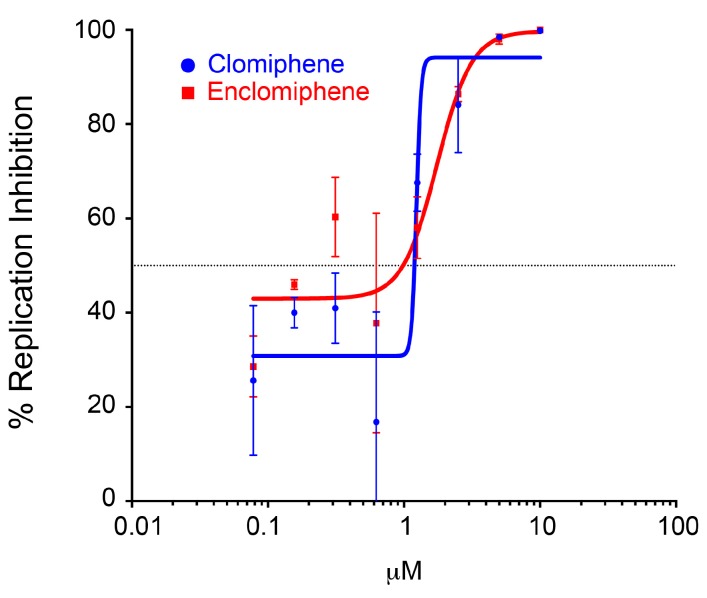
Clomiphene and enclomiphene display similar dose responses for blocking trVLP infection. Target HEK293T/17 cells, prepared as for Figure 1, were pretreated for 1 h at 37 °C, and then infected in the presence of increasing concentrations of clomiphene or enclomiphene (serial 2-fold dilutions from 10 μM). 48 h later, they were assayed for trVLP infection as described in the legend to Figure 1. Data ± SD are from triplicate samples normalized to the average value from mock treated samples (cells only background subtracted).

**Figure 4:**
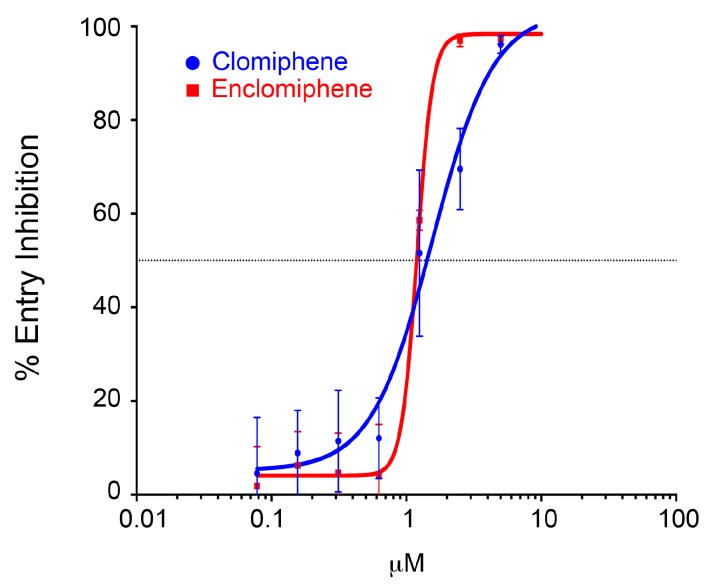
Clomiphene and enclomiphene display similar dose responses for blocking EBOV VLP entry. HEK293T/17 cells were pretreated with increasing concentrations of clomiphene or enclomiphene (serial two-fold dilutions from 10 μM) for 1 h at 37 °C. VLPs bearing EBOV GP (Mayinga strain) were then bound to the cells in the presence of the indicated concentration of drug and cytoplasmic entry was assayed as described in the legend to Figure 2. Data ± SD are from triplicate samples normalized to the average % entry in mock treated cells (41.8%).

**Figure 5:**
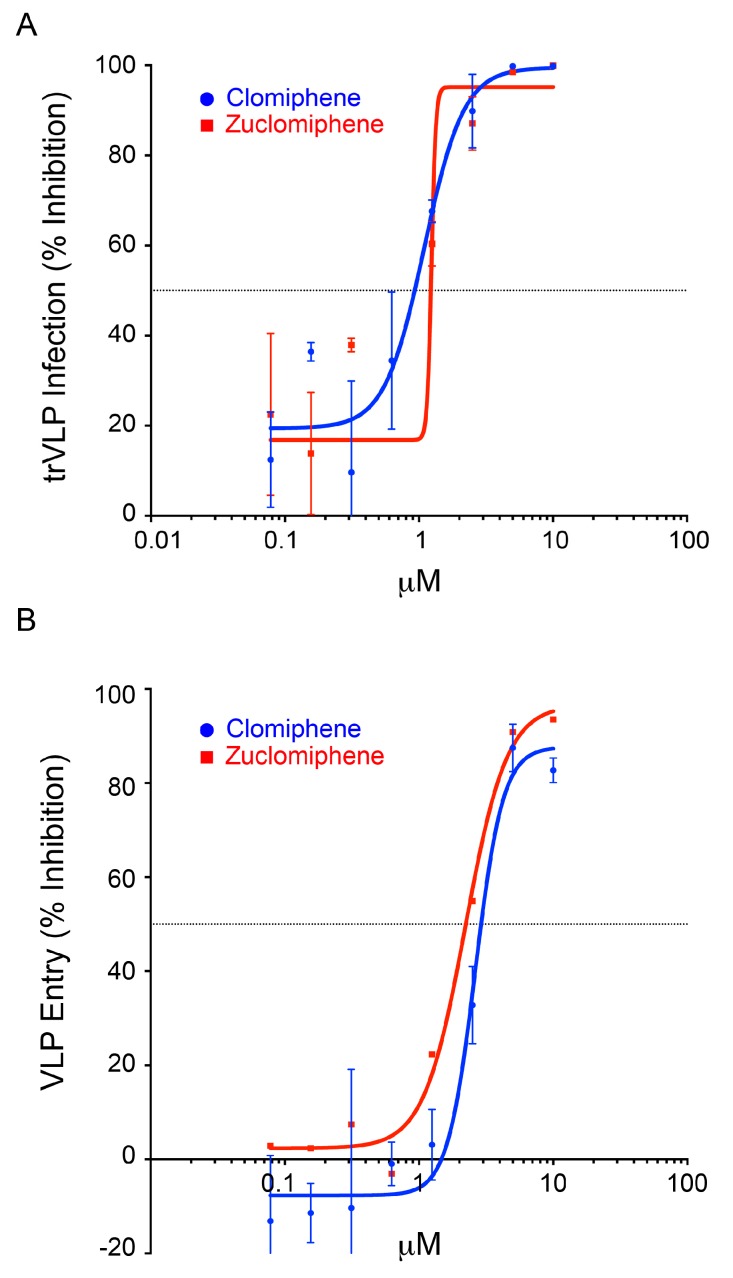
Clomiphene and zuclomiphene display similar dose responses for blocking EBOV trVLP infection and EBOV VLP entry. (**A**) Target HEK293T/17 cells were prepared as described in the legend to Figure 1, and were treated with clomiphene or zuclomiphene and infected, as described in the legend to Figure 3. Data ± SD are from triplicate samples normalized to the average value from mock treated samples; and (**B**) HEK293T/17 were seeded, treated with clomiphene or zuclomiphene, and VLPs bearing EBOV GP (Mayinga strain) were bound to the cells as described in the legend to Figure 4. Cytoplasmic entry was assayed as described in the legend to Figure 2. Data ± SD are from triplicate samples normalized to the average % entry in mock treated cells (15.6% and 19.7% for the two sets of drugs).

We first compared clomiphene and enclomiphene in the trVLP infection and VLP entry systems using eight-point dose response curves. As seen in Figure 3, clomiphene and enclomiphene showed similar dose-dependent inhibition of trVLP infection. In this experiment the calculated IC_50_ for clomiphene was 1.2 μM and that for enclomiphene was 1.0 μM. Clomiphene and enclomiphene also showed similar dose-dependent inhibition in the VLP entry system; the IC_50_ for clomiphene was 1.4 μM and for enclomiphene was 1.2 μM (Figure 4). Similar results were seen when comparing eight doses of clomiphene and zuclomiphene in trVLP infection (Figure 5A) and VLP entry (Figure 5B) assays. The IC_50_ and IC_90_ values from all of the eight-point dose response curves (Figure 3, Figure 4 and Figure 5) are given in Table 1.

The observed IC_50_ values (in HEK 293T/17 cells) are in good agreement with each other for clomiphene vs. enclomiphene and for clomiphene vs. zuclomiphene and for the trVLP infection vs. VLP entry assays. Moreover, they are in range with the IC_50_ values calculated for the effect of clomiphene on EBOV infection of Vero E6 cells; 2.42 μM for GFP-encoding and 3.83 μM for native EBOV (Mayinga) [20].

### 3.3. Enclomiphene Blocks trVLP Infection in Vero E6 Cells as well as Entry Mediated by the Makona EBOV and Angola MARV GPs

**Figure 6:**
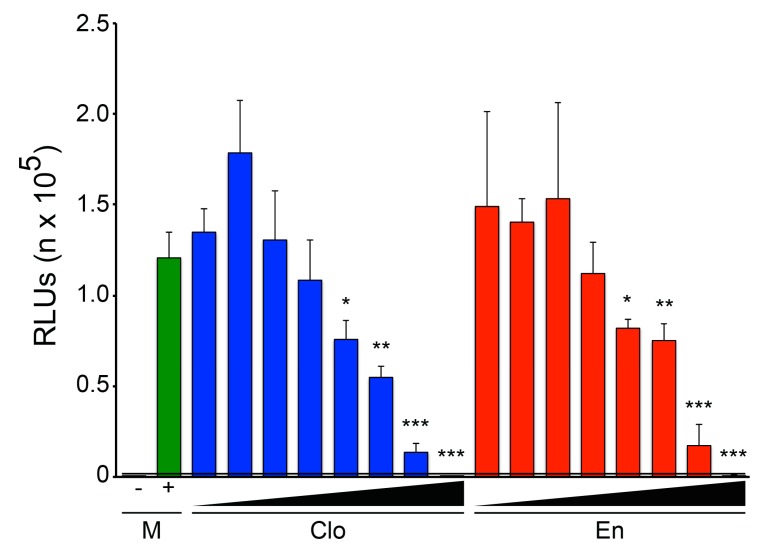
Clomiphene and enclomiphene block trVLP infection in Vero E6 cells. 18–24 h after transfection (as for Figure 1) Vero E6 cells were pretreated with DMSO (mock, M) or increasing amounts (serial two-fold dilutions from 10 μM) of Clo or En. After 1 h at 37 °C, the cells were infected in the presence of drug and trVLP infection assayed as for Figure 1. Data are averages of triplicate samples (cells only background subtracted) ± SD. The black bar and line indicate background signal (−, cells transfected with GFP in place of EBOV L). Significance relative to mock: * *p* ≤ 0.05, ** *p* ≤ 0.01, *** *p* ≤ 0.001. Zuclomiphene was not tested in this experiment.

Previous work using authentic EBOV in a BSL4 lab showed that clomiphene blocks infections in multiple cell types including Vero E6 (kidney), HepG2 (liver), and HUVEC (endothelial) cells, and by all strains of filoviruses tested: three strains of Ebola virus and two of Marburg virus [20,21]. Here we used assays that can be conducted in a BSL2 laboratory to begin to test whether enclomiphene shows similar breadth as a filovirus inhibitor. We first used the trVLP system to compare the efficacy of clomiphene and enclomiphene in Vero E6 cells. The trVLP experiments presented thus far were conducted in HEK 293T/17 cells, which are optimal for this assay because they are highly transfected with the multiple plasmids needed to produce trVLPs and to assay their lifecycle capacity [42,43]. Vero E6 cells are also good targets for this assay. As seen in Figure 6, clomiphene and enclomiphene blocked trVLP infection in Vero E6 cells (with similar dose profiles).

**Figure 7:**
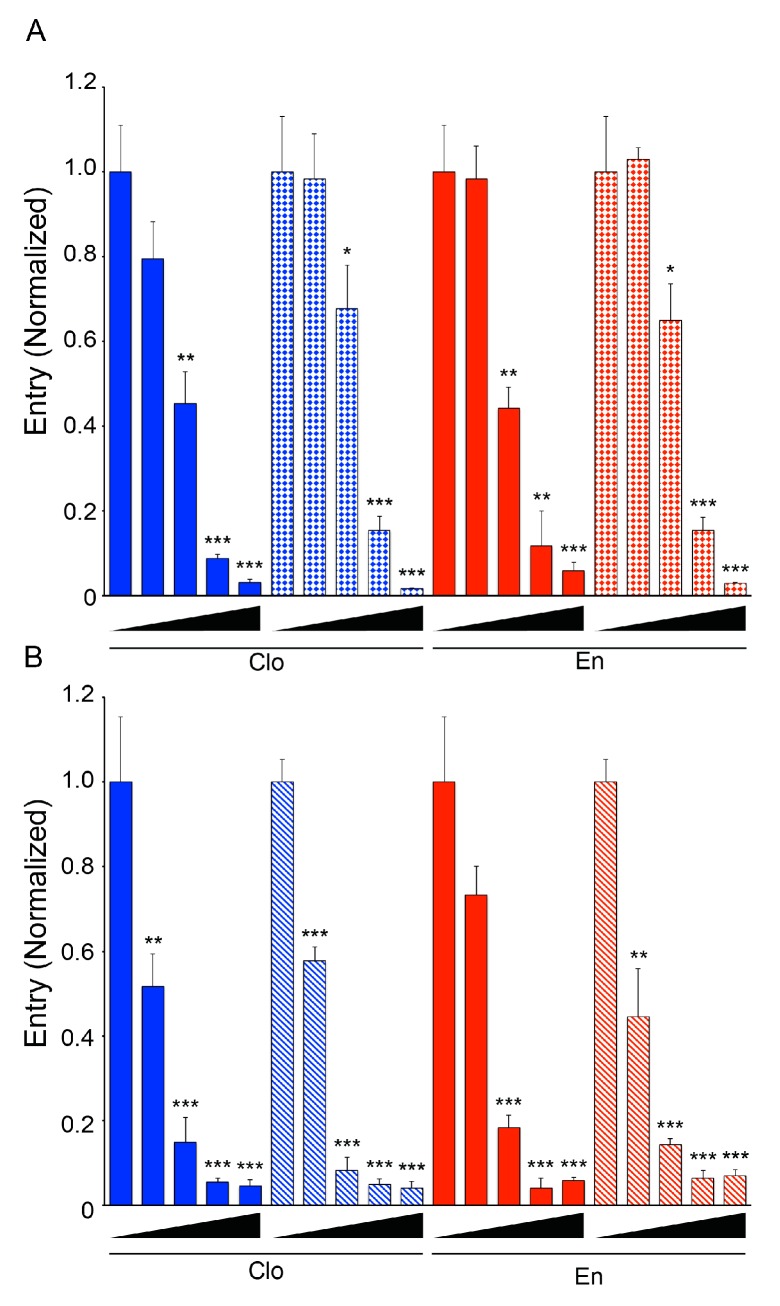
Clomiphene and enclomiphene block entry of VLPs bearing GPs from other filoviruses. Vero E6 cells were treated with 0, 1, 2.5, 5, or 10 μM of Clo or En and incubated for 1 h at 37 °C. VLPs bearing (**A**) EBOV GP Mayinga (solid) or EBOV GP Makona (grid), (**B**) EBOV GP Mayinga (solid), or MARV Angola (diagonal) were bound to the cells in the presence of Clo (blue) or En (red) and cytoplasmic entry was assayed and analyzed as described for Figure 2. Data ± SD were normalized to the average % entry for mock treated cells incubated with the same VLP type (A: EBOV Mayinga, 51.6%; EBOV Makona, 51.8% and B: EBOV Mayinga, 16.1%; MARV Angola, 17.9%). Asterisks indicate significant inhibition of entry relative to mock (DMSO) treated samples (* *p* ≤ 0.05, ** *p* ≤ 0.01, *** *p* ≤ 0.001). Zuclomiphene was not tested in these experiments.

The current trVLP system utilizes plasmids encoding proteins from the Mayinga (1976) isolate of EBOV [42,43], an oft-used reference strain. We, therefore, utilized entry reporter VLPs, which can accommodate glycoproteins from other viruses [20,29], to ask whether the ability of enclomiphene to block EBOV entry extends to other filoviruses. As seen in Figure 7A, enclomiphene exerted a similar effect as clomiphene in blocking entry mediated by the GP from Makona EBOV, the isolate that caused the 2014–2015 outbreak in Western Africa, and, as seen in Figure 7B, enclomiphene inhibited entry mediated by a Marburg virus GP, representing a different genus within the filovirus family, similar to the effects of clomiphene.

## 4. Discussion

Clomiphene emerged from two independent screens of FDA-approved drugs for anti-EBOV activity in tissue culture cells [20,21,22], and was also found to provide 60%–90% protection in female mice challenged with a lethal dose of EBOV [20,21]. Further analyses demonstrated that clomiphene blocks EBOV entry into the host cell cytoplasm after virus particles are transported to endolysosomes [20,29], the site of EBOV fusion and cytoplasmic entry [30,31,32]. Hence, clomiphene interferes with the process of EBOV fusion with the endolysosomal membrane.

Clomiphene, as used to treat female infertility, is a ~60:40 mixture of two stereoisomers, enclomiphene and zuclomiphene. Since zuclomiphene is thought to impart more side effects [40], enclomiphene is being developed for the treatment of secondary hypogonadism (low testosterone) [41]. The main objective of this study was to assess the abilities of enclomiphene and zuclomiphene to block EBOV infection, as the individual isomers might offer (different) advantages in different clinical settings. We found that enclomiphene and zuclomiphene were highly similar to clomiphene in their ability to block trVLP infection and VLP entry, which are validated surrogate assays for EBOV infection and EBOV entry, respectively. Clomiphene, enclomiphene, and zuclomiphene inhibited VLP entry and trVLP infection with similar IC_50_ values. Where tested, they were equally effective to each other in two cell types and for entry mediated by three filoviral GPs. 4-hydroxy-enclomiphene, and 4-hydroxy-zuclomiphene also blocked EBOV trVLP infection and VLP entry under the conditions tested. Therefore, we propose that, like clomiphene, enclomiphene and zuclomiphene have potential as pan-filoviral inhibitors in multiple cell types.

Approximately 11,000 individuals died during the recent outbreak of Makona EBOV, and another ~16,000 have survived their infection [1]. Tragically, many of these survivors are displaying post Ebola virus disease syndromes, including visual problems, hearing loss, and excessive fatigue [3,4]. In addition, infectious EBOV has been found in semen for up to six months [5], raising concerns of sexual transmission [6]. Hence, drugs that accumulate in tissues of the eye and male reproductive tract could be particularly helpful for certain Ebola virus survivors. In this respect it is interesting that, in mice, both enclomiphene and zuclomiphene persist in the eye and male reproductive tract longer than in other tissues, with zuclomiphene maintained at higher levels than enclomiphene. Zuclomiphene was also found to persist in the brain, whereas enclomiphene was not [39].

Clomiphene provided up to 90% protection in the lethal mouse model of EBOV infection when administered to female mice at a dose of 60 mg/kg on days 0, 1, 3, 5, 7, and 9 post-infection, with no apparent ill effects on surviving mice. The maximum serum concentration (C_max_) for a 60 mg/kg dose (intra-peritoneal administration, IP) of clomiphene to female mice was 5.1 μM (Lisa Johansen, personal communication) and, hence, above the IC_50_ for blockade of EBOV infection in tissue culture cells [20,21] and above the IC_50_ values reported here for clomiphene and its isomers in blocking EBOV VLP entry and trVLP infection (Table 1). The standard dose of clomiphene to treat female infertility is 50 mg/day (per os). With this (oral) dose, reported C_max_ values (summed for enclomiphene and zuclomiphene) range from 0.04 to 0.05 μM (Table 2), ~50–60-fold below the IC_50_ (2.4 μM in Vero E6 cells) for anti-EBOV activity [20,21]. Therefore, for potential treatment of acute EBOV infections, higher doses of clomiphene would certainly be needed, likely in combination with other anti-EBOV drugs. The human equivalent dose (to the mouse dosing employed in [20]) for an adult weighing 60 kg would be 292 mg clomiphene every other day [46]. In this respect it is noteworthy that higher doses of clomiphene (up to 250 mg/day) have been administered to non-responding (anovulating) females [47,48], and that considerably higher C_max_ values were obtained when patients were administered the standard dose of clomiphene (50 mg), but by intravenous administration (IV) instead of the oral route (Table 2) [49]. Hence, it is conceivable that higher doses of clomiphene could be administered (perhaps as part of a cocktail) to treat acute EBOV infections.

Irrespective of the utility of clomiphene for acute EBOV infections, given the persistence of the clomiphene isomers in the eye and male reproductive tract [39], clomiphene (alone or in a combination) may have utility in the management of post Ebola virus disease symptoms in EBOV survivors. For example, enclomiphene and zuclomiphene accumulate ~50- to ~150-fold, respectively, in the Harderian gland (located in the orbit) and the uveal tract of the mouse eye. Similar accumulation of zuclomiphene was seen in tissues of the male mouse reproductive tract [39]. If such accrual occurs in humans, the standard dose (50 mgs per day, per os) of clomiphene, which contains both isomers, could be sufficient to reach anti-viral levels in the eye and male reproductive tract; the levels would more likely be sufficient if clomiphene were given at a higher dose and/or in combination with another anti-EBOV agent. Interestingly, no sperm were seen in the testes and epididymes of male mice treated for 90 days with 40 mg/kg/day of zuclomiphene [40].

Since the two isomers of clomiphene and their hydroxylated metabolites all blocked EBOV VLP entry and replication in cell cultures, the protective effect of clomiphene in the mouse model [20,21] could have been due to any or all of these forms. The metabolism of en- and zuclomiphene in humans is well-characterized [37,38], and differences between the isomers have been shown in mice [39,40]. It will, therefore, be interesting to compare the efficacies of the individual clomiphene isomers in the mouse model of EBOV infection. Studies on their relative efficacies in the eyes and reproductive tract will be especially revealing.

## 5. Conclusions

We have shown that, like the parent mixture, the two isomers of clomiphene (and their hydroxylated metabolites) block entry and replication of EBOV VLPs in cell cultures. The activity of the isomers was shown in two cell types and against three filoviral GPs. Both isomers of clomiphene accumulate in tissues of the eye and male reproductive tract. Hence we propose that clomiphene, an FDA-approved drug that provided up to 90% protection in the mouse model of Ebola virus disease, remain in consideration as an anti-EBOV agent, especially for the management of post Ebola virus disease syndromes, including visual problems and the risk of sexual transmission, and especially for consideration as part of a potential drug cocktail.

## Figures and Tables

**Table 1:** Comparison of IC_50_ and IC_90_ values for clomiphene and its isomers in trVLP infection and VLP entry assays.

Drug	Assay	Set 1		Set 2	
		IC_50_ (μM)	IC_90_ (μM)	IC_50_ (μM)	IC_90_ (μM)
Clomiphene	trVLP	1.2	1.4	0.9	2.2
	Entry	1.4	4.1	2.9	4.4 *
Enclomiphene	trVLP	1.0	2.8		
	Entry	1.2	1.6		
Zuclomiphene	trVLP			1.2	1.4
	Entry			2.2	5.6

Set 1 values are from experiments that compared clomiphene with enclomiphene (Figure 3 and Figure 4), and Set 2 values are from experiments that compared clomiphene with zuclomiphene (Figure 5). * EC_90_: GraphPad did not compute an IC_90_ value. For all other values, the IC_50_ vs. EC_50_ as well as IC_90_ vs. EC_90_ values were within 93% ± 6% (SD) of each other. From the two datasets the average values for clomiphene were: IC_50_ trVLP, 1.0 μM; IC_90_ trVLP, 1.8 μM; IC_50_ Entry, 2.1 μM; IC_90_ Entry, 4.2 μM. In all cases, the ratio of IC_90_/IC_50_ was ≤2.9.

**Table 2:** Review of data on pharmacokinetics of clomiphene and its isomers in humans

Compound Administered	Compound Analyzed	Dose (mg) ^a^	Route	Sex	Status	Ave. of n	C (max) (μM)	AUC (ng/mL/h) (last time)	T (max)	T 1/2 (h)	Ref.
Clomiphene		50	Oral	F	Healthy	24					[37]
	Enclomiphene ^b^						0.02	42 (24 h)	3.7	---	
	Zuclomiphene ^b^						0.02	662 (288 h)	6.8	---	
Clomiphene		50	Oral	F	Anov.	9					[45]
	Enclomiphene						0.01	65 (72 h)	3	---	
	Zuclomiphene						0.04	1289 (456 h)	7	---	
Clomiphene		50	IV	F	Anov.						[49]
	Enclomiphene					N/A, P1	0.66	373 (24 h)	---	60	
						N/A, P2	1.21	862 (24 h)	---	283	
	Zuclomiphene					N/A, P1	0.15	134 (24 h)	---	341	
						N/A, P2	0.26	255 (24 h)		802	
Enclomiphene	Enclomiphene ^c^	25	oral	M	ITT	16	0.04	---	2.5	---	[41]

**^a^** Once per day; **^b^** Data are from Table 3 in reference [45]; **^c^** Values deduced from Figure 4 of reference [41]; Anov., anovulating; h, hour; N/A, not applicable; P1, anovulatory patient 1; P2, anovulatory patient 2; ITT, intention-to-treat (i.e., healthy males with low testosterone levels).
